# Sustaining Home Medical Care in a Mountainous Rural Region: A Qualitative Study of Physician Motivation, Burden, and Nighttime Care Systems

**DOI:** 10.7759/cureus.103219

**Published:** 2026-02-08

**Authors:** Kazuya Maeda, Ryosuke Hase, Yuta Horinishi, Ryuichi Ohta

**Affiliations:** 1 Family Medicine, Yamaguchi University Graduate School of Medicine, Ube, JPN; 2 Public Health and Preventive Medicine, Yamaguchi University Graduate School of Medicine, Ube, JPN; 3 Community Care, Unnan City Hospital, Unnan, JPN

**Keywords:** after-hours care, family, family medicine, general medicine, home care services, physicians, primary health care, qualitative research, rural health services

## Abstract

Introduction: As population aging accelerates, demand for home medical care is increasing, particularly in rural and mountainous regions where access to hospital-based services is limited. However, sustaining home care, especially nighttime and out-of-hours coverage, remains challenging due to workforce constraints and the accumulated burden on physicians. This study examined how the perceived “attractiveness” and “burdens” of home medical care are linked to the sustainability of nighttime care systems in a rural Japanese context.

Methods: This qualitative study integrated autoethnography describing the first author’s experience with urban home medical care and semi-structured interviews with physicians practicing home care in Unnan City, a mountainous rural area in Japan. Interview transcripts and field notes were analyzed using inductive qualitative content analysis, with triangulation across data sources to identify recurring themes related to motivation, burden, and system sustainability.

Results: Five interrelated themes were identified: a sense of mission to deliver care to patients unable to access hospitals; substantial nighttime and out-of-hours burden experienced as geographical strain, emotional labor, and persistent tension; intrinsic enjoyment derived from engaging with patients’ everyday lives; professional fulfillment associated with providing seamless care across emergency, inpatient, outpatient, and home settings; and educational value for younger physicians. Importantly, participants described that the nighttime burden could be mitigated through sufficient staffing and rotation-based duty distribution, and that continuity of care enhanced both job satisfaction and training opportunities.

Conclusions: The interaction between professional meaning, workload distribution, and system design shapes the sustainability of rural home medical care. Beyond a narrative of sacrifice, rural home care can be sustained when meaningful clinical work, fair allocation of nighttime duties, and educational opportunities are aligned to support workforce retention and continuity of care.

## Introduction

Population aging in Japan is rapidly increasing the demand for home-based medical care [1,2]. Although national surveys consistently show that a majority of citizens prefer to spend the end of their lives at home, the proportion of deaths occurring at home remains low, with substantial regional variation [3,4]. Previous studies have suggested that dying at home may be associated with better quality of death, higher satisfaction among patients and families, and reduced caregiver burden [5,6]. Therefore, enabling individuals to choose their preferred place of care at the end of life is not only a matter of healthcare access but also a critical issue related to the realization of personal values and autonomy in the final stage of life.

In sparsely populated rural areas, however, the provision of home medical care is structurally disadvantaged due to long travel distances and limited healthcare resources [7,8]. In Japan, institutional frameworks such as home care support clinics and hospitals, including function-enhanced models, have been established to support 24-hour availability, after-hours home visits, and end-of-life care, and their presence has been associated with higher rates of nighttime visits and home deaths [9]. Nevertheless, in rural and mountainous regions, limited workforce capacity and weak inter-organizational networks often make it difficult to meet the requirements for these function-enhanced models, resulting in their concentration in urban areas [10,11].

Workforce constraints further threaten the sustainability of rural home care. The average age of clinic-based physicians in Japan is 60.4 years, rising to 62.4 years in Shimane Prefecture [12]. A prefectural survey has projected a decline in the number of clinic physicians by 2029, along with reductions in nighttime home visits and a growing shortage of successors [13]. These trends raise serious concerns about the sustainability of routine outpatient services and home medical care, particularly in rural settings that require after-hours and on-call responses.

Nighttime and on-call duties have been reported to be associated with decreased work motivation, burnout, and increased risks to patient safety [14]. Accordingly, discussions of sustainable home care in rural areas must go beyond professional values or a sense of mission alone. It is essential to examine how nighttime responsibilities are operationally distributed among physicians, how burdens are shared or concentrated, and how local resource configurations, such as the availability of visiting nurses as first responders, can mitigate physician workload [15].

Against this background, Unnan City Hospital, a public hospital situated in a mountainous rural area of Shimane Prefecture, has played a central role in providing outpatient, inpatient, and home medical care as part of a comprehensive care model that spans both hospital and home settings [16]. While public hospitals with generalist departments often serve as key providers of home care in rural regions, there is limited empirical research describing how physicians themselves experience this work [17,18]. In particular, little is known about how physicians perceive the attractiveness of home care practice (e.g., professional fulfillment and educational value), the burdens they experience (e.g., nighttime duties, geographic constraints, psychological tension), and how these experiences relate to the recruitment, retention, and development of younger physicians and the sustainability of after-hours care systems.

Therefore, this study aims to explore the meanings physicians attribute to their work in rural home medical care and to examine how these meanings relate to the sustainability of the local healthcare workforce and care delivery systems. Using a qualitative design that integrates semi-structured interviews with physicians at Unnan City Hospital and the first author’s autoethnographic reflections, this study seeks to elucidate the interplay between perceived attractiveness, experienced burdens, and the long-term viability of rural home care in a resource-constrained setting.

## Materials and methods

Objectives

Drawing on prior clinical experience in home medical care, it became evident that the sustainability of 24-hour home care systems was strongly influenced by contextual factors, including the availability of local healthcare and long-term care resources, population density, travel distance, and institutional support structures. Against this backdrop, the present study aimed to examine how home medical care, including end-of-life care at home, was realized in rural and mountainous areas, and under what conditions it could be sustained. Using Unnan City, Shimane Prefecture, as a case study, this research explicitly sought to clarify how physicians engaged in home medical care interpreted and gave meaning to their practice under geographic and institutional constraints; how work patterns and role allocation, including nighttime and after-hours responsibilities, related to the sustainability of home care delivery; and how these practices interacted with the surrounding community and institutional environment [19,20]. Through a qualitative analysis integrating semi-structured interviews with physicians and the first author’s autoethnographic reflections, this study further aimed to identify key elements necessary for building sustainable home medical care systems in rural settings.

Study design and setting

The study was conducted at Unnan City Hospital, located in Unnan City, Shimane Prefecture, Japan. This study employed a qualitative research design to explore home medical care practices in rural Japan, integrating autoethnography and semi-structured interviews. In addition, the first author undertook a one-month field stay in Unnan City, observing outpatient consultations and home visits to understand local healthcare resources and cultural context, and documented these observations as field notes. Autoethnography was used as a reflexive data source to contextualize and contrast urban and rural home care systems, while interviews with rural physicians were conducted to capture lived experiences and locally grounded practices [21].

Autoethnography

The first author worked as a registered nurse for five years in the emergency department and intensive care unit of a tertiary hospital after graduating from university. During this period, the author became aware of the importance of advance care planning prior to hospitalization and subsequently engaged in home nursing practice. This included experience in visiting nursing, on-site management, and organizational administration. To enhance patients’ quality of life at the end of life, the author also established a private, self-funded service aimed at supporting patients’ self-realization [21].

To further support patients’ goals of care from a medical perspective, the author later enrolled as a medical student at a national university medical school and is currently in training. Drawing on these experiences as a healthcare professional, service administrator, and medical student, the autoethnographic component provided reflective descriptions of the institutional, cultural, and practical characteristics of urban home medical care. These reflections were used as an analytical reference point rather than as stand-alone empirical data [21]. No formal coding or direct comparison with rural interview data was conducted; instead, the autoethnographic accounts were used reflexively to sensitize interpretation and contextualize differences in care structures between urban and rural settings.

Interviews with rural physicians

Participants and Sampling

Participants were selected using purposive sampling. Eligible participants were physicians practicing in Unnan City, Shimane Prefecture, who met the following criteria: ongoing clinical practice within Unnan City; current involvement in home medical care; and experience managing at least five home care patients within the previous year. Physicians who met these criteria were invited to participate, and written informed consent was obtained after providing detailed explanations of the study’s purpose, methods, and ethical considerations.

Data Collection

Semi-structured interviews were conducted face-to-face in Japanese. Each interview lasted approximately 60 minutes. Prior to participation, all interviewees received an explanation of the study and provided consent for audio recording. All interviews were recorded and transcribed verbatim for analysis. The interview guide was developed based on insights derived from the autoethnographic reflections on urban home care and covered five domains: organization and resources of home medical care delivery; characteristics and needs of patients and families; interprofessional collaboration; relationships with the local community and cultural context; and perceived differences between urban and rural home care settings.

Data analysis

Interview transcripts were analyzed using an inductive qualitative content analysis approach [22]. Initial codes were generated inductively through repeated, line-by-line reading of transcripts, with meaning units identified based on physicians’ descriptions of experiences, perceptions, and evaluations of rural home medical care. Codes were iteratively compared across transcripts to identify similarities and differences, and were refined through ongoing analytic memo-writing.

During the coding process, preliminary codes and memos were shared with co-authors, and regular analytic discussions were held to examine how well codes reflected the data, to consider alternative interpretations, and to refine code definitions. Codes with conceptual similarity were grouped into higher-order categories, and candidate themes were developed to capture patterned relationships among categories. These themes were repeatedly reviewed against the full dataset to ensure coherence, internal consistency, and coverage across participants.

Findings from the autoethnographic component were not coded or treated as primary data. Instead, autoethnographic insights were introduced reflexively during analytic meetings as a contrastive and contextual lens. For example, observations from urban home care practice were used to question whether interpretations of rural nighttime burden, role allocation, or continuity of care reflected contextual constraints rather than universal features of home medical care. In this way, autoethnography contributed to theme refinement and interpretive depth by challenging initial assumptions and sharpening analytic distinctions, while the interview data remained the principal analytic foundation.

Throughout the analysis, discrepancies in coding or interpretation were addressed through reflexive dialogue among the research team rather than forced consensus. Themes were iteratively revised through multiple rounds of discussion to enhance credibility and trustworthiness, ensuring that final interpretations were grounded in the interview data while informed by diverse professional perspectives.

Ethical considerations

This study was approved by the Clinical Ethics Committee of Unnan City Hospital (approval number: 20250007). All participants were informed orally and in writing about the study objectives, procedures, voluntary nature of participation, and data usage, and written informed consent was obtained. All transcripts and autoethnographic descriptions were anonymized to prevent identification of individuals or institutions. Field notes from the observational component were likewise anonymized, and no patient-identifying information was recorded.

## Results

Autoethnography: comparison of home medical care delivery systems in urban and rural settings

In this study, the first author’s autoethnographic accounts were used to describe structural differences in home medical care delivery systems between urban and rural settings. For analytical comparison, six categories were defined: clinical style and care processes; role allocation and relationships with patients; modes of travel and home visit implementation and associated workload; nighttime and emergency response systems; information-sharing mechanisms and medical technology infrastructure; and pharmacy collaboration and sustainability of resources. Below, characteristics of urban and rural home medical care systems are presented in a contrasting manner across these categories.

Urban home medical care delivery system

Clinical Style and Care Processes

Urban home medical care was characterized by high efficiency and systematic operation. Nurses conducted thorough pre-visit preparation, while physicians visited patients’ homes sequentially, spending approximately three to five minutes per patient in a high-turnover, efficiency-oriented model. Allocation of visits was relatively random and coordinated by care managers, and it was not considered problematic for a different physician to complete death certification during nighttime hours.

In contrast, rural home medical care was centered on relationships with patients and their families. Physicians routinely engaged in conversations about daily life and personal circumstances before clinical examination, confirming contextual information, including life history and values. Background information about patients and families was often shared across generations, and physicians visited homes with an understanding of each household’s living conditions. During critical moments such as end-of-life care, there was a clear expectation that the primary physician would be present at the time of death, in contrast to the urban model, where physician substitution was institutionally accepted.

Role Allocation and Relationships With Patients

In urban settings, roles among healthcare professionals were clearly delineated. Nurses performed the most direct patient interventions, including vital sign measurements, blood sampling, and physical care. Physicians conducted brief interviews, auscultation, and prescription decisions based on nurses’ reports and completed documentation on-site. As a result, the time physicians and patients spent face-to-face was structurally limited.

In rural settings, role allocation was more fluid. Physicians frequently performed basic procedures themselves, including vital sign checks and blood sampling. During visits, physicians were often invited into the home and engaged in consultations while seated among family members. Casual conversation and emotional support were provided with the same intensity as physical examinations. Physicians were perceived not merely as medical providers but as figures closely embedded in family life, and the depth of these relationships was described as a source of professional fulfillment.

Modes of Travel, Home Visit Implementation, and Workload

In urban areas, home visits were conducted by three-person teams consisting of a driver, nurse, and physician. Visits followed tightly scheduled itineraries, enabling efficient coverage of geographically proximate households. High daily visit volumes (approximately five to 10 visits per day) were planned, and time management constituted a core operational element.

In rural areas, visits were often conducted by a physician alone or by a physician and nurse, with minimal staffing. Visits were typically scheduled between outpatient clinic sessions, covering a small number of patients per round. Due to wide geographic dispersion, travel times were long, and limited public transportation meant that many patients were unable to attend clinics independently, resulting in frequent, unavoidable home visits. Clinical documentation was often completed after visits, sometimes using a combination of electronic and paper records. This structure prioritized accessibility over efficiency, increasing physicians’ physical workload.

Nighttime and Emergency Response Systems

In urban settings, nighttime and after-hours responsibilities were distributed across multiple mechanisms. Small clinics managed on-call duties internally, while larger clinics employed dedicated nighttime physicians who covered home visits and end-of-life care through shift-based systems. These large providers also contracted with other institutions to outsource on-call coverage, thereby reducing emergency department utilization at the community level. Additionally, external emergency home-visit services specializing in nighttime care were available, ensuring institutionalized access to after-hours support.

In rural areas, nighttime home visit systems were less stable. Particularly in clinics operated by a small number of physicians, emergency calls from terminally ill or pre-terminal patients tended to concentrate during nighttime hours, requiring individual physicians to respond personally to late-night requests. Consequently, nighttime and holiday duties were indispensable for enabling patients to remain at home until death, yet simultaneously constituted the most physically and emotionally exhausting aspect of physicians’ work.

Information-Sharing Systems and Medical Technology Infrastructure

In urban areas, electronic medical records were seamlessly shared between home-visit sites and clinics, with systems that enabled on-site prescription printing. Cloud-based platforms facilitated time-independent information sharing among visiting nurses, home care workers, and pharmacies. Furthermore, home medical devices, such as patient-controlled analgesia (PCA) pumps, were widely available and managed competently across professions, enabling home pain control comparable to hospital standards.

In rural areas, information sharing relied heavily on interpersonal and individualized communication, with continued dependence on paper documents and fax transmission. Real-time electronic documentation during visits was often impractical, resulting in delayed record entry. Devices for home-based pain control, such as PCA pumps, were less widely available, and some nurses had limited experience in their use. These limitations contributed to anxiety regarding pain management and acute deterioration, potentially influencing patients’ and families’ decisions to opt for hospitalization despite a preference to remain at home.

Pharmacy Collaboration and Resource Sustainability

In urban areas, high patient density within limited geographic zones supported the economic feasibility of visiting pharmacy services, enabling regular medication delivery and adherence support. Such collaboration served as critical infrastructure for medication adjustments at night and on weekends, ensuring continuity of home-based palliative care. Many urban settings were also staffed predominantly by younger physicians, facilitating rotation-based coverage for nighttime and emergency duties.

In rural areas, patient households were widely dispersed, making it challenging to sustain pharmacy services financially. Prescriptions were often not dispensed on-site during home visits, instead requiring family members to collect medications later from clinics or pharmacies. Consequently, continuity of home care depended heavily on families’ mobility and self-management capacity. Barriers to timely medication access, particularly during terminal phases or acute deterioration, emerged as structural factors influencing the feasibility of home-based end-of-life care.

Interview analysis results

Theme Identification

We qualitatively analyzed verbatim transcripts from semi-structured interviews (I1-I12) together with verbatim field-note records, coding and comparing meaning units. I1-I12 indicate individual semi-structured interviews with participating physicians (Interviewee 1 to Interviewee 12). L1-4, L7-12, etc. refer to the corresponding line numbers in the verbatim interview transcripts. This process yielded five overarching themes: a sense of mission as providers of rural healthcare, nighttime/out-of-hours burden and sustainability, interest and joy in supporting daily living, the appeal of providing seamless care across hospital and home, and educational value. These themes provide an analytic framework illustrating how physicians structured their lived experiences, values, and perceived challenges in rural home care. Representative excerpts (with interview and line numbers) and their implications are presented below (Table 1).

**Table 1:** Themes and excerpts related to motivation, challenges, and expectations in rural home care I1–I12 indicate individual semi-structured interviews with participating physicians (Interviewee 1 to Interviewee 12). L1–4, L7–12, etc. refer to the corresponding line numbers in the verbatim interview transcripts.

Theme	Illustrative excerpts (source)
Sense of mission as providers of rural healthcare	“The number of patients we can see is small, and it’s not efficient.” (I11, L1–6) / “Many people in this area can’t come to the hospital. So going to see them is simply our role—we can’t stop.” (I12, L1–4,14–16) / “Delivering medical care to those who cannot travel is the role of a community clinic physician.” (I12, L14–15) / “If we don’t take this on, there’s nowhere else to go—this is effectively the last resort.” (I9, L4–7) / “Even if outpatient care is ideal, many patients have difficulty visiting.” (I1, L6–10) / “Six to seven in ten want home care, but fewer than one in ten die at home.” (I3, L7–9) / “Only about 10–20% die at home; supply shortages are a factor.” (I4, L4–4)
Nighttime/out-of-hours burden and sustainability	“In the past, out-of-hours home visits were common. All urgent calls went to community clinic physicians.” (I12, L7–12) / “Nighttime care at the end of life is exhausting.” (I4, L15–21) / “When they call, you have to go—and it’s tough to absorb all their anger and anxiety.” (I6, L3–10) / “Going on a home visit deep into the mountains at midnight is tiring.” (I7, L1–6) / “Before a patient dies, there’s a constant tension—you might be called every day.” (I8, L1–10) / “There aren’t many midnight calls, but before death you might be called at 2 a.m. We have enough staff to rotate and share it.” (I10, L18–22) / “Now it’s maybe once a month at most; with enough people, it’s not a major burden.” (I11, L4–5) / “Before, one person carried the night phone for a whole week; now we can distribute it through a rota.” / “Keeping on-call responsibility outside work hours feels heavy.” (I7, L22–26)
Interest and joy in supporting daily living	“It’s interesting to enter people’s living spaces and provide care within the family circle.” (I1, L11–15) / “I want to respond to the community, and I keep doing this because I like it.” (I3, L5–5) / “It’s really enjoyable to provide care almost like a family member and to be consulted.” (I1, L2) / “I continue because I like it—I want to support people’s lives.” (I4, L6–8) / “When patients return home, they look calmer and more energetic.” (I3, L7–8) / “Home visits feel like being invited into the patient’s territory—it refreshes me too.” (I5, L1–2) / “Seeing their lives makes me want to do my best, and I like going around the community.” (I8, L1–4) / “Because it’s their home, you can see the whole family dynamic—that’s fascinating.” (I7, L1–5) / “You can become closer than on the ward; at home you notice things you otherwise wouldn’t.” (I10, L2–6) / “It’s good that you can talk at length without being constrained by time.” (I10, L6–8)
Appeal of seamless care across the hospital and home	“You can work across the ER, wards, home visits, and outpatient care.” (I1, L1–8) / “It matters to care for someone you met in the ER during admission and then continue after they return home—through to the end.” (I3, L5–5) / “Continuity from emergency care to admission to home is very meaningful.” (I3, L5–5,11–11,14) / “A strength is that you can switch from outpatient care to home visits (and back).” (I1, L6–7) / “Even as a home-visit physician you can arrange necessary tests and admissions.” (I4, L12–15) / “After seeing the home situation, it’s helpful that the same team can care through admission and death.” (I5, L1–9) / “I can do everything I want to do.” (I9, L1–5) / “Ideally the same physician would care through death, but we can’t fully achieve that yet.” (I6, L1–2) / “It’s not boring because you can do emergency, outpatient, inpatient, and home visits.” (I10, L6–12) / “It’s interesting to follow the patient all the way through.” (I11, L2–4) / “In Unnan, you can do ER, wards, home visits, and outpatient care—it’s really enjoyable.” (I1, L5)
Educational value	“Continuous care from emergency care to admission to home is educationally meaningful.” (I3, L5) / “As a resident, experiencing end-of-life care for terminal cancer was truly valuable.” (I4, L22–23) / “Home-visit cases become part of residents’ portfolios and discussion material.” (I5, L1–9) / “This rural training lets you explore where you want to place your professional focus.” (I2, L1–6) / “Even if trainees struggle in one area, there’s another path forward.” (I6, L1–2,18) / “You see things differently at first contact versus on the ward.” (I10, L12–18) / “Following the same patient from emergency to home stops you imposing ideal theories.” (I9, L1–6) / “You can experience field-specific issues such as opioid use at end of life.” (I11, L3–4)

Sense of Mission as Providers of Rural Healthcare

In the present data, physicians acknowledged that “the number of patients we can see is small, and it’s not efficient” (I11, L1-6). Despite this premise, they articulated a strong sense of regional responsibility and fulfillment of patient needs, stating that “many people in this area cannot come to the hospital. So going to see them is simply our role, it’s something we cannot stop doing” (I12, L1-4, 14-16), and that “even if outpatient care is ideal, there are many patients who have difficulty visiting” (I1, L6-10).

Furthermore, physicians described their role as a last resort, noting that “if we don’t take this on, there’s nowhere else for them to go, this is effectively the final option” (I9, L4-7). They also highlighted a gap between preference for home care and actual home death, stating that “six to seven out of ten people wish to receive care at home, but fewer than one in ten actually die at home” (I3, L7-9), and that “only about 10-20% die at home, and a lack of service supply is a contributing factor” (I4, L4-4).

Nighttime and Out-of-Hours Burden and Sustainability

Clinic-based physicians described that, historically, “there were many out-of-hours home visits, and all emergency calls were concentrated on community clinic physicians” (I12, L7-12), reflecting the burden of small-scale staffing. At present, hospital-based physicians reported that “to be honest, nighttime care during the terminal phase is exhausting” (I4, L15-21), indicating a heavy nighttime burden in end-of-life care.

At the same time, several physicians noted a reduction in burden through task distribution, stating that “with more staff, the workload can be shared through rotation” (I10, L18-22), that “previously, one person carried the night phone for an entire week, but now it’s distributed through an on-call rotation” (I9, L1-5), and that “now it happens maybe once a month at most; because there are enough people, it’s not a major burden” (I11, L4-5).

In addition, physicians described emotional labor, such as “when they call, you have to go, and you also have to absorb their anger and anxiety” (I6, L3-10), as well as persistent tension, exemplified by “before a patient dies, there’s a constant feeling that you might be called every day” (I8, L1-10), and “keeping on-call responsibility outside working hours feels psychologically heavy” (I7, L22-26). They also referred to geographical burden, stating that “going on a home visit deep into the mountains late at night is exhausting” (I7, L1-6).

Interest and Joy in Supporting Daily Living

Regarding home medical care, physicians described a sense of closeness to family life and intimacy, noting that “it’s interesting to enter people’s living spaces and provide care within the family circle” (I1, L11-15), and that “it’s enjoyable to provide care and consultations from a position almost like a family member” (I1, L2).

As reasons for continuing this work, they stated that “I continue because I like it-I want to support people’s lives” (I4, L6-8), “I also like going around the community” (I8, L1-4), and “because it’s their home, you can see the entire family relationship, and that’s fascinating” (I7, L1-5), reflecting the enjoyment and refreshment derived from supporting daily life. One physician commented that “home visits feel like being invited into the patient’s territory, and it’s refreshing for me as well” (I5, L1-2).

Regarding patient changes, physicians observed emotional improvement at home, stating that “when patients return home, their expressions become calmer and they regain energy; seeing that makes me feel it was worth doing” (I3, L7-8). They also described deeper insights compared with ward care, noting that “there are discoveries you can only make at home” and that “it’s good to be able to talk slowly and in depth without being constrained by time” (I10, L2-6, L6-8).

Appeal of Providing Seamless Care Across Hospital and Home

Within the study setting (the catchment area of Unnan City Hospital), a system was observed in which the same physician or a small team provided continuous emergency, inpatient, outpatient clinic, and home visit care. Physicians described that “continuity from emergency care to admission to home care is meaningful” (I3, L5-5, 11-11, 14), and that “after seeing the home situation, it was reassuring that the same team could continue care even after admission, all the way through end-of-life care” (I5, L1-9), indicating cross-phase continuity across the entire care process.

At the same time, physicians pointed out a gap between ideals and reality, stating that “ideally, the same physician would provide care from outpatient visits through to end-of-life, but at present we cannot fully achieve that” (I6, L1-2). In operational terms, they described flexibility in care settings, such as “the ability to switch back and forth between outpatient care and home visits depending on the patient’s condition” (I1, L6-7), and comprehensive continuity, noting that “even as a home-visit physician, I can handle necessary examinations and arrange hospital admission myself” (I4, L12-15).

Physicians also expressed high job satisfaction and intellectual stimulation, stating that “it’s interesting to be able to follow a patient all the way through” (I11, L2-4), that “I’m allowed to do everything I want to do” (I9, L1-5), and that “being able to do emergency care, outpatient clinics, inpatient wards, and home visits keeps the work interesting and not monotonous” (I10, L6-12).

Educational Value

Physicians further described the educational value of continuous care, stating that “continuous care from emergency care to admission and then to home care is educationally meaningful” (I3, L5). Younger physicians reported learning experiences unique to the field, such as “being able to experience end-of-life care for terminal cancer patients was truly valuable” (I4, L22-23), and “I could experience issues that only arise in practice, such as opioid use at the end of life” (I11, L3-4).

Home care cases were also described as being utilized as educational resources, with one physician noting that “home-visit cases are incorporated into residents’ portfolios and used as material for discussion” (I5, L1-9). Through movement across multiple care settings, physicians reported acquisition of diverse perspectives, stating that “following the same patient from emergency care to home prevents you from imposing idealistic theories on other settings” (I9, L1-6), and that “the way you see things differs between the initial encounter and the ward, and you come to understand people’s feelings better” (I10, L12-18).

Additionally, within the training process, physicians described opportunities for career orientation, such as “being able to explore where you want to place your professional focus” (I2, L1-6), and identified a safety-net function, noting that “even if you struggle in one area, there is another place where you can find a way forward” (I6, L1-2, 18).

## Discussion

This study integrated the first author’s autoethnography on home medical care practice in an urban setting with semi-structured interviews with physicians in Unnan City, Shimane Prefecture, to examine how the “attractiveness” and “burdens” that support the continuation of home visits in a mountainous rural region are linked to the sustainability of nighttime care systems. The main findings were: a normative commitment to delivering care to people who cannot easily access hospital services, together with practices that engage deeply with patients’ everyday lives, functioned as key drivers of home visits; nighttime and out-of-hours duties were experienced as geographical travel burdens, emotional labor, and persistent tension, while staffing scale and rotation systems could distribute these burdens; and continuity across emergency care, hospitalization, outpatient care, and home care was described as both clinical fulfillment and educational value and may contribute to maintaining the system by facilitating the entry and retention of younger physicians.

First, physicians in Unnan interpreted home visits as a “role” that cannot be explained by efficiency alone. This resonates with prior literature suggesting that rural home visits may be framed as “obligation” or responsibility in remote settings [23]. A distinctive feature of the present study is that this norm was not limited to expressions of devotion; rather, engagement with the life context of patients and families itself was described as a “felt sense of work” and professional satisfaction [24]. This aligns with reports that primary care physicians derive meaning through experiences of meaningful work [25]. In other words, the continuation of home visits may be experienced not only as “what must be done,” but also as “what one wants to do” (enjoyment, relationships, and the tangible sense of supporting daily life) [26].

Second, nighttime and out-of-hours duties were described as a central burden shaping sustainability. Previous studies have reported that on-call burden may be associated with burnout, intention to leave, and risks to patient safety [27]. The present study adds value by specifying this burden as “geographical travel” (long-distance travel late at night), “emotional labor” (absorbing anxiety and anger), and “persistent tension” (anticipatory standby before a death). Importantly, physicians suggested that the same nighttime duty can shift from a condition in which the burden is concentrated to one in which it is distributed, depending on staffing scale and rotation design. Accordingly, sustainability in Unnan can be understood not only as value-driven but also as shaped by operational conditions, including rotation design (frequency, coverage, fairness) and team configuration [2].

Third, the experience of continuously caring for patients across emergency care, hospitalization, outpatient care, and home care was described not only as clinically satisfying but also as educationally valuable. A key implication of this study is that rural home care should not be framed solely in terms of “burden.” Instead, continuity of care may serve as a learning opportunity and be linked to the entry and retention of younger physicians, which in turn connects to the distribution of nighttime burden and system maintenance [28]. This relationship can be summarized as a circular hypothesis: attractiveness of continuity of care (clinical fulfillment/educational resource) → entry and retention of younger physicians → increased staffing → more distributed rotation coverage → reduced nighttime burden → sustained service delivery. However, this cycle is likely to require training designs that include adequate rest protection and supervisory structures.

For future consideration, remote areas may find it challenging to meet urban-oriented institutional requirements in the same way [29]. In such contexts, an alternative model worth examining is one in which hospitals and clinics collaborate to consolidate workforce capacity and share nighttime duties through a rotation system. Furthermore, considering evidence that visiting nurses may serve as first responders for out-of-hours contact, the sustainability of nighttime responses may depend not only on distributing the physician on-call burden, but also on the extent to which mitigation strategies, such as information sharing, interprofessional coordination, and information and communication technology (ICT) infrastructure, can be implemented alongside first-response functions by visiting nursing services [30]. Therefore, the effectiveness of rotation design should be evaluated in conjunction with system conditions (staffing, role-allocation rules, and the availability of first-response resources).

This study has several limitations. This study used semi-structured interviews with a small number of physicians engaged in home medical care in Unnan City and the first author’s autoethnography and examined the coherence of narratives regarding continuity across emergency care, hospitalization, outpatient care, home care, and end-of-life care; nighttime burden; and educational significance, positioning this as part of data triangulation. However, systematic member checking was not conducted; therefore, participants did not have the opportunity to formally review or validate analytic interpretations, which may limit confirmability and interpretive robustness. In addition, the short-term and cross-sectional nature of data collection and incomplete theoretical saturation limit the extent to which the durability of younger physicians’ “enjoyment,” their retention motivation, the effectiveness of burden-mitigation strategies, and the stability of the proposed process model can be fully ensured.

Furthermore, given the specific context of Unnan City, this study does not claim generalizability beyond the national level. Rather, key components, such as consolidation of nighttime support, ICT-enabled information sharing, stable deployment of visiting nursing services, and access to pain management resources, should be treated as comparative reference points. Credibility was instead supported through iterative analytic discussions among multiple authors, reflexive examination of alternative interpretations, and triangulation of interview findings with autoethnographic insights. Validity and testability could be strengthened through follow-up interviews, participant feedback, comparative case studies across other regions, cross-checking by multiple analysts, and the development of an auditable trail.

## Conclusions

This study indicates that the sustainability of home medical care in mountainous rural regions depends not solely on physicians’ sense of obligation but on the interaction between professional meaning, operational burden, and system design. In Unnan City, home visits were sustained by a normative commitment to reaching patients who cannot access hospital care and by the intrinsic satisfaction of engaging with patients’ everyday lives, while nighttime and out-of-hours duties posed the greatest threat to sustainability through geographical strain, emotional labour, and persistent tension. Importantly, these burdens were not fixed; when staffing levels and rotation systems enabled a fair distribution of night duties, individual burdens were reduced. Furthermore, continuity of care across emergency, inpatient, outpatient, and home settings was experienced as clinically and educationally valuable, suggesting that rural home care can attract and retain younger physicians and, through increased staffing, indirectly support the stabilization of nighttime care systems. Overall, the findings suggest that sustainable rural home medical care should be understood not as a model of sacrifice, but as an integrated system in which meaningful work, distributed responsibility, interprofessional collaboration, and supportive training structures together enable continuity of care in remote and aging communities.
